# Evaluation of an implementation support package to increase community mental health clinicians’ routine delivery of preventive care for multiple health behaviours: a non-randomised controlled trial

**DOI:** 10.1186/s43058-023-00509-0

**Published:** 2023-11-13

**Authors:** Casey Regan, Kate Bartlem, Caitlin Fehily, Elizabeth Campbell, Christophe Lecathelinais, Emma Doherty, Luke Wolfenden, Richard Clancy, Marcia Fogarty, Agatha Conrad, Jenny Bowman

**Affiliations:** 1 School of Psychological Sciences, The University of Newcastle, Callaghan, NSW 2308 Australia; 2 Hunter New England Population Health, Locked Bag 10, Wallsend, NSW 2287 Australia; 3 The Australian Preventive Partnership Centre (TAPPC), Sax Institute, Ultimo, NSW Australia; 4 Hunter Medical Research Institute, Kookaburra Circuit, New Lambton Heights, NSW 2305 Australia; 5 School of Medicine and Public Health, The University of Newcastle, Callaghan, NSW 2308 Australia; 6 School of Nursing and Midwifery, The University of Newcastle, Callaghan, NSW 2308 Australia; 7 Hunter New England Mental Health Services, Po Box 833, Newcastle, NSW 2300 Australia; 8 Central Adelaide Local Health Network, PO Box 17, Fullarton, SA 5063 Australia

**Keywords:** Preventive care, Mental health service, Smoking, Nutrition, Alcohol, Physical activity, Implementation strategies

## Abstract

**Background:**

People with a mental health condition are more likely to engage in risk behaviours compared to people without. Delivery of preventive care to improve such behaviours is recommended for community mental health services, but inadequately implemented. This study assessed the effectiveness of an implementation support package on clinicians’ delivery of preventive care (assessment, advice, referral) for four risk behaviours (tobacco smoking, harmful alcohol consumption, physical inactivity, inadequate fruit and vegetable intake) compared to no implementation support. The participatory approach to developing the support package, and fidelity of the implementation strategies, are also described.

**Methods:**

A non-randomised controlled trial was undertaken in 2019–2020 with two community mental health services (control and target) in one health district in New South Wales, Australia. A 4-month support package consisting of multiple implementation strategies was delivered to one site following a two-phase participatory design process. Five implementation strategies were proposed to service managers by researchers. After consultation with managers and clinicians, the final implementation support package included four strategies: training and education materials, enabling resources and prompts, client activation material, and audit and feedback. Client-reported receipt of the three elements of preventive care for the four risk behaviours was collected from a cross-sectional sample of clients who had recently attended the service at baseline (6 months) and follow-up (5 months). Logistic regression models examined change in receipt of preventive care to assess effectiveness.

**Results:**

A total of 860 client surveys were completed (control baseline *n* = 168; target baseline *n* = 261; control follow-up *n* = 164; and target follow-up *n* = 267). Analyses revealed no significant differential changes in preventive care receipt between the target and control sites from baseline to follow-up, including across the four primary outcomes: assessed for all behaviours (OR = 1.19; 95% CI 0.55, 2.57; *p* = 0.65); advised for all relevant risk behaviours (OR = 1.18; 95% CI 0.39, 3.61; *p* = 0.77); referred for any relevant risk behaviour (OR = 0.80; 95% CI 0.40, 1.63; *p* = 0.55); and complete care (OR = 3.11; 95% CI 0.62, 15.63; *p* = 0.17). Fidelity of the implementation strategies was limited as one of the four strategies (audit and feedback) was not delivered, components of two strategies (enabling resources and prompts, and client activation material) were not delivered as intended, and one strategy (education and training) was delivered as intended although some components were offered late in the implementation period.

**Conclusions:**

The implementation support package was ineffective at increasing preventive care delivery. Further investigation is required to determine optimal participatory design methods to develop effective implementation strategies, including those that support delivery of care in community mental health settings within the ongoing context of uncertain environmental challenges.

**Trial registration:**

Australian and New Zealand Clinical Trials Registry ACTRN12619001379101.

**Supplementary Information:**

The online version contains supplementary material available at 10.1186/s43058-023-00509-0.

Contributions to the literature

- Preventive care for tobacco smoking, poor nutrition, harmful alcohol use and physical inactivity is not routinely delivered by mental health clinicians to service consumers.
- Implementation strategies can support mental health services to provide preventive care.
- An implementation support package was developed utilising a participatory design approach with clinicians and managers from a community mental health service; however, it was not effective at increasing preventive care receipt.
- The trial was conducted during the 2020 wave of the COVID-19 pandemic which significantly impacted the service.
- Further research is needed to design effective implementation strategies that support preventive care delivery within the ongoing context of COVID-19.

## Introduction

Globally, people with a mental health condition experience greater morbidity and mortality due to chronic disease compared to the general population [1, 2]. Tobacco smoking, poor nutrition, harmful alcohol use and physical inactivity are modifiable health risk behaviours associated with chronic disease development [3–6] and are more prevalent among people with a mental health condition compared to the general population [1, 7–9]. Although people with a mental health condition are interested in modifying these behaviours, they may need additional support to do so [10–13]. Mental health services are well placed to provide such support due to the routine nature and frequency of contact, and established rapport between client and clinician [14–19].

The delivery of preventive care (routine assessment and management of risk behaviours) by health care providers is an evidence-based intervention to improve health behaviours [20–22]. Clinical practice guidelines recommend the systematic provision of preventive care during routine health service appointments [23, 24]; including within mental health services [25, 26]. An endorsed approach to providing preventive care is the ‘5As’ model: ‘ask’ about behaviours, ‘assess’ interest in change, provide ‘advice’ to change, provide behaviour change ‘assistance’, and ‘arrange’ referral [24, 27]. To overcome barriers such as time constraints, the abbreviated ‘AAR’ model (assess, advise, and refer) is recommended at a minimum [28, 29] and has demonstrated effectiveness [30], including among clients attending mental health services [31]. Despite the evident potential for mental health services to provide preventive care, systematic review evidence demonstrates that preventive care is infrequently provided in mental health services internationally [10, 20, 32], and there is a need to develop and implement strategies to support services in providing care.

Systematic review evidence shows there have been few trials assessing the effectiveness of implementation strategies to support mental health clinicians to provide preventive care [9]. The review identified 20 studies that assessed the impact of any strategy to increase at least one component of the 5As for any of the four key risk behaviours. Fourteen were found to be effective in increasing at least one element of preventive care provision. Effective strategies included clinician training, educational materials, health information systems, local consensus processes, authority and accountability, and reminders.

A previous study by the research team was the only study identified in the review and the only study the authors could locate, that aimed to increase care for all four behaviours in routine mental health appointments [32]. The multiple baseline trial assessed the effectiveness of six implementation strategies in increasing AAR throughout 12 community mental health services. Client-reported receipt of preventive care before and after the implementation support package increased for only one of 16 outcomes. An evaluation of the same implementation strategies in the same health district but within generalist community health services resulted in increased assessment and advice outcomes [33, 34]. The difference in effectiveness between the two studies may have been due to the implementation strategies being generic, and not adequately addressing the clinical, professional, and organisational factors that are specific to the community mental health settings. This indicates a need for future research to develop and test implementation strategies to increase preventive care that do appropriately consider the context of community mental health service delivery.

Participatory approaches provide opportunities to include key stakeholders in design, maximising the potential that implementation strategies are suitable and meet the needs of end-users [35, 36]. Involving mental health clinicians in the development of implementation strategies to support practice change has been suggested to enhance the likelihood of strategies being acceptable and aligning with the context [37]. Several implementation trials in mental health settings which have involved mental health clinicians in strategy design have demonstrated improved clinical practices (e.g. increased conversations about smoking [38] and metabolic syndrome screening [39]), and improved client outcomes (e.g. increased primary care access [40] and reduced cigarette consumption [38]).

This study aimed to assess the effectiveness of an implementation support package on community mental health clinicians’ routine delivery of preventive care (assessment, advice and referral) for four health risk behaviours (tobacco smoking, harmful alcohol consumption, physical inactivity and inadequate fruit and vegetable intake) compared to no implementation support. The participatory approach and fidelity of the implementation strategies were also described.

## Methods

### Study design and setting

A non-randomised controlled trial was undertaken with two community mental health services that provide care to adult clients with a mental health condition (one control site and one target site) in one health district in New South Wales (NSW), Australia. In Australia, community mental health services are typically government-funded services and provide early diagnosis, acute clinical care, and rehabilitation to clients with a range of serious mental health diagnoses (e.g. schizophrenia, severe depression or anxiety, and comorbid substance use). These services are the most accessed specialty mental health service in Australia [41]. The health district in which the study was conducted has a policy requiring clinician assessment, advice and referral to ongoing behaviour change support services for clients with chronic disease risk behaviours, including smoking, inadequate fruit and vegetable consumption, physical inactivity, and harmful alcohol consumption [42]. Based on consultation with the district’s Mental Health Executive Director, community mental health services within the district were considered eligible based on geographical feasibility for the research team delivering the support strategies, and services with equivalent staff sizing. Of the four services meeting these criteria, two were excluded due to involvement in similar research, the remaining service in closest geographical proximity to the research team was allocated as the target site, and the other as the control site.

Following a two-phase participatory design process (details provided below), a four month support package consisting of multiple implementation strategies aiming to support clinicians in the delivery of preventive care was undertaken with the target site (November 2019 to February 2020). Outcome measurement included telephone surveys conducted with cross-sectional samples of clients at baseline over six months (26 April 2019 to 17 October 2019) and follow-up over five months (12 May 2020 to 14 October 2020).

The first COVID-19 wave in Australia began to emerge during the delivery of the implementation strategies (January 2020), with state-wide lockdowns occurring at the end of the implementation period (March 2020) and continuing until the third month of the follow-up data collection period.

The four primary outcomes for the study were client-reported receipt of assessment of risk status for all four health behaviours; advice for all risks for which they were assessed (for those with at least one risk); referral offer for at least one risk for which they were assessed (for those with at least one risk); and complete care (assessment of all behaviours, advice for all risks, referral for at least one risk). Secondary outcomes were client receipt of assessment, advice and referral for each of the four behaviours (tobacco smoking, alcohol consumption, physical activity and fruit and vegetable intake (12 outcomes)).

The trial was approved by the Hunter New England Human Research Ethics Committee (Approval No. 18/11/21/4.06) and the University of Newcastle Human Research Ethics Committees (Approval No. H-2019–0108). Reporting is in accordance with the Standards for Reporting Implementation Studies (StaRI) Statement. The trial was registered with the Australian and New Zealand Clinical Trials Registry (ACTRN12619001379101).

### Participants and recruitment

#### Eligibility

At baseline, adult clients of both sites were eligible to be recruited for data collection if they: had attended at least one individual in-person appointment within the previous 4 months and had a phone number listed in the health district’s electronic medical record. At follow-up, eligibility criteria were adjusted to also include telehealth appointments within the previous four months, due to service changes in appointment types as a result of COVID-19 restrictions. Clients were not excluded from follow-up data collection if they had been selected for, or participated in, the survey at baseline. At both time-points, additional eligibility criteria were assessed on contact by trained interviewers: English speaking, mentally and physically capable of responding to survey items, and not currently living in aged care facilities or gaol.

#### Recruitment

Potentially eligible clients were identified via electronic medical records. We aimed to sample approximately *n* = 600 clients (*n* = 300 per site) at baseline and at follow-up to have 80% power to detect a 14% increase in assessment and a 19% increase in advice and referral outcomes [32, 43]. For both the target and control sites, approximately 30 clients were randomly selected each week across both sites during the baseline and follow-up periods. Clients were mailed an information sheet informing them of the survey and data collection procedures. They were provided with a toll-free number they could call if they did not wish to be contacted for participation. Clients were contacted approximately two weeks later via telephone by trained interviewers employed by the health service and asked whether they would like to participate. If clients identified as being of Aboriginal and/or Torres Strait Islander origin, they were offered the opportunity to have their interview conducted by an Aboriginal interviewer. Participants could withdraw from the study during the survey, and participation in data collection and receipt of care were independent.

### Target condition

The Community Mental Health Service allocated to the target site received a four month implementation support package aiming to increase their provision of preventive care to clients using the AAR model of care. The service employed 47 clinical staff at the start of the trial (including full and part-time staff equivalent to approximately 21 full-time staff members), and 46 at the end of the trial, with 39 staff being employed for the full 4 months. The implementation support strategies included within the trial were designed with target site staff, during the six months prior to implementation. The model of care and implementation support package (including the participatory approach) are outlined below.

### Model of preventive care

The intervention was clinician provision of the preventive care model ‘Assess, Advise, Refer’ (AAR), in line with the health district’s preventive care policy. The policy directs clinicians to offer clients an assessment of four health risk behaviours (tobacco smoking, inadequate fruit and vegetable consumption, physical inactivity, and harmful alcohol consumption). Clients identified as being at risk as defined by Australian national guidelines and recommendations [44–47] for any of the health risk behaviours are: provided brief advice on the benefit of to changing their behaviours to meet the Australian national guidelines; and, offered a referral to specialised behaviour change support services (e.g. evidence-based, state-wide telephone support services New South Wales (NSW) Quitline for smoking [48], the NSW Get Healthy Information and Coaching Service for inadequate fruit and vegetable intake, inadequate physical activity and harmful alcohol consumption [49], or a local general practitioner (GP), or allied health provider).

### Implementation support package

The initial selection of implementation strategies was informed by implementation research and review evidence [9, 50–53]. Five implementation strategies were identified by researchers as potentially suitable to trial in the community mental health service and were proposed to managers and clinicians: (1) champion/local opinion leaders; (2) clinician educational meetings/training and educational materials; (3) enabling resources and prompts for clinicians (staff activation); (4) client activation materials; and (5) audit and feedback (see Table 1). The components and contents of the strategies were modified according to input and feedback provided by clinical managers and clinicians over a two-phase participatory design process that occurred over seven months. This allowed the implementation strategies proposed initially to be refined to suit the context of the community mental health care service.

**Table 1:** Implementation support package: strategy proposed in the participatory design process, description of the final strategy for inclusion, and delivery to service

Strategy proposed and suggestions	Description of strategy to be included	Week^a^ delivered to intervention service^b^
***(1) Champion/local opinion leaders***		
*Proposed:* Designated mental health support ‘champion’ is recruited from within the service to promote the role of mental health clinicians’ in providing preventive care *Feedback:* Phase one of the participatory design process identified this strategy as not being appropriate due to the service manager wanting all staff to share an equal role in preventive care delivery	Not included after phase one of the participatory design process	Not included
***(2) Clinician educational meetings/training and educational materials***		
*Proposed:* An education and training package will provide evidence regarding the relevance of each behavioural risk factor to the mental health and wellbeing of clients (in addition to their physical health), as well as address key clinician barriers to preventive care provision, such as misperceptions about client disinterest in changing behaviours and receiving preventive care *Feedback:* During phase one of the participatory design process, the implementation working group (service manager, clinical coordinator, psychiatrist, dietitian, senior mental health nurse, and the research team) identified training sessions and materials to be useful	**Training sessions (sub-strategy 1):** Six 45-minute training sessions were designed and delivered over six weeks (delivered once^a^ or twice) to cover six topics: (A) preventive care; (B) smoking cessation; (C) physical activity and nutrition; (D) motivational interviewing; (E) the Get Healthy Telephone Coaching Service; and (F) alcohol reduction. Session recordings were made available via Youtube link. Training contents, topics, and delivery frequency and timing were informed by clinician requests generated during phase two. Training sessions were delivered over a series of interactive workshops during the service’s existing time slots for educational meetings. Training sessions focused on specific behaviours (B, C and F) provided education on the links between such behaviour and mental health outcomes, examples of questions to ask to assess, and examples of referral avenue. The motivational interviewing training session provided guidance to clinicians on how to provide behaviour change assistance to clients, including, for example, strategies to keep smoking on the agenda, utilising OARS (open-ended questions, affirmation, reflective listening, summarise), scaling, and eliciting change talk (desires, abilities, reasons and needs)	(A) 1st (*N* = 28)^c^ (B) 2nd (*N* = 30) (C) 3rd (*N* = 29) (D) 4th (*N* = 28) (E) 5th (*N* = 19) (F) 6th (*N* = 23)
	**Educational booklets (sub-strategy 2):** Four types of clinician educational resources booklets (one for smoking (A), nutrition (B), physical activity (C), and alcohol (D)) were developed and 200 × printed copies were provided to the service (50 copies for each behaviour). Booklet contents were informed by suggestions generated by clinicians during phase two. For example, guidance for clinicians on how to assist clients make behaviour changes was provided, including conversation starters, brief and achievable tips and strategies to change, motivational interviewing guidance with example phrases, visual aids and common misconceptions. Electronic copies of each booklet were made available online via the service’s network drive. Delivery of booklets occurred at multiple timepoints during the 4-month period	A: 7th B: 8th C: 8th D: 10th
***(3) Enabling resources and prompts for clinicians (staff activation)***		
*Proposed:* Enabling resources including educational information/materials for clinicians to provide to clients, paper-based assessment tools, clinical decision-making supports, referral forms and prompting posters *Feedback:* During phase one of the participatory design process, the implementation working group suggested enabling resources and prompts would be helpful. Clinicians requested referral forms and client resources to be placed in each consultation room for ease of access	**Clinician assessment tool (sub-strategy 3)**^**d**^**:** A tool for clinicians to assess client risk for all behaviours was adapted from a previously existing tool to be briefer and more client focused. Clinicians provided extensive input on multiple iterations of the assessment tool 50 × paper-based assessment tools were provided **Referral forms (sub-strategy 4)**^**d**^**:** Paper-based referral forms for NSW Get Healthy Service and Quitline were sourced and provided to the service. 50 × GHS and Quitline referral forms were provided **Client resources (sub-strategy 5)**^**d**^**:** Paper-based resources that could be given to clients by clinicians were sourced and provided to the service. This included: 350 × brochures of seven types (50 per type) including one for each of the four behaviours, a Get Healthy Telephone Coaching Service brochure, an alcohol-focused Get Healthy Telephone Coaching Service brochure and a smoking Quitline brochure; 50 × smoking information packs termed a ‘QuitKit’; and paper-based behaviour information leaflets (‘client handouts’) **Clinician poster (sub-strategy 6)**^**d**^**:** A poster was designed and placed in the staff meeting room to prompt clinicians and managers to provide preventive care and discuss preventive care in review meetings	Sub-strategy 3: 15th Sub-strategy 4: 12th Sub-strategy 5: 7th, 12th Sub-strategy 6: 12th
***(4) Client activation materials***		
*Proposed:* These strategies are employed to engage the clients to be more proactive regarding asking for and receiving preventive care support from the clinicians *Feedback:* Client activation materials were appealing to members of the implementation working group, who suggested materials could be placed in the waiting room	**Conversation cards (sub-strategy 7)**: Coloured conversation starter cards (four types for each behaviour) were placed in the waiting room by researchers. Forty coloured conversation starter cards were provided (10 for each behaviour) **Client poster (sub-strategy 8)**: A poster was designed and placed in the client waiting room to prompt clients to ask their clinician about SNAP behaviours in upcoming appointments **Client self-assessment form (sub-strategy 9)**: A double-sided, 10-item, paper-based form for clients to self-assess risk for all behaviours was designed and provided to the waiting room reception. 50 × printed pre-appointment forms (plus 10 × clip boards and pens) were provided. Clinicians suggested the assessment form for clients to fill in waiting room. The form was reviewed by a consumer representative for appropriateness and clarity	Sub-strategy 7: 7th Sub-strategy 8: 12th Sub-strategy 9: 16th
***(5) Audit and feedback***		
*Proposed:* Tailoring of the locally prescribed generic preventive care assessment tool for mental health services which is revised based upon clinicians’ feedback and clinical relevance for the sessions with clients. Then feedback is provided to the staff members about the current preventive care progress of their clients may be incorporated on a regular basis during the staff meetings with the clinical manager *Feedback:* The audit and feedback strategies were primarily developed with implementation working group during Phase one, and refined during additional meetings with the team leader	Two streams of audit and feedback were identified as appropriate in the participatory design process: during client-focused 13-week review meetings (team leader or clinical co-ordinator reviews progress over last 13 weeks for 40 clients) (sub-strategy 10) and during clinician-focused caseload reviews (team leader or clinical co-ordinators reviews each clinician’s caseload with clinician every 13 weeks) (sub-strategy 11) **Sub-strategy 10:** Discussion from the client-focused 13-week review meetings are typed up as notes in the clinical review template. Preventive care discussion was to be recorded in the ‘summary’ section **Sub-strategy 11**: A tailored and brief preventive care assessment tool to capture AAR for each *snap* behaviour would be incorporated into the existing template used for each client during the clinician caseload reviews	Not delivered Although identified as appropriate by staff, both audit and feedback sub-strategies were unable to be implemented due to service changes, including reformatting of clinical case review forms, and COVID-19 limitations, including the significant reduction of review meetings to avoid face-to-face contact and align with COVID-19 protocols

^a^Week of the intervention period (16 weeks in total from the start of November 2019 to the end of March 2020, excluding the last week of December 2019 and the first 3 weeks of January 2020)

^b^To comply with the COVID-19 hygiene protocol taking effect during the final week of the intervention period, shared materials (e.g. conversation starter cards) were removed from the waiting room and staff were advised to avoid handing clients with any hardcopy materials (e.g. brochures)

^c^Number of staff attendees

^d^Electronic master versions of all resources were transferred to the service’s network drive

#### Participatory design process: phase one

An initial implementation working group (including the service manager, clinical coordinator, psychiatrist, dietitian, senior mental health nurse, and the research team) met six times over a five month period prior to the implementation support package commencement. The working group discussed whether and how the five proposed implementation strategies might offer potential solutions to current challenges in delivering preventive care. Such challenges reported in the literature, and relevant to the service, include a lack of resources, time, skills, and perception of client motivation. Four of the five proposed implementation strategies were perceived as applicable (excluding champions/local opinion leaders) to address these barriers (Table 1). There were always at least three service staff represented at each of the six meetings. Usual support was provided to clinicians regarding preventive care delivery from the service manager during phase one.

#### Participatory design process: phase two

All service clinicians were provided the opportunity to participate. At commencement of phase two, clinicians were informed by service management about the upcoming implementation support package and the outcomes of the implementation working group meetings in phase one. Over two months, all clinicians were invited to three meetings that occurred during their regular morning in-service meeting session. The purpose of these sessions was to introduce the four implementation strategies as planned at the end of phase one and to provide clinicians opportunities to offer input regarding their content and delivery.

The care delivery model was augmented, at the request of clinicians, to include resources to support clinicians to ‘Assist’ client behaviour change (in line with the 5As model; though not assessed as an outcome of the study). For example, the resource booklets provided education on motivational interviewing tips with examples of phrases that clinicians might use, as well as brief, practical tips clinicians might offer clients to assist behaviour change.

#### Final implementation support package

The research team finalised the implementation strategies based on input from phases one and two and provided the implementation working group with a final set of strategies for approval. Project personnel (program manager and PhD student) supported delivery of the implementation support package and provided ongoing assistance as needed or requested through face-to-face visits with managers and clinicians, and phone calls and/or e-mails to managers. See Table 1 for a description of strategies proposed during the participatory design process, and a full description of the final strategies included. The final implementation support package was based on four of the five strategy categories initially proposed in phase one and included 11 component sub-strategies (Table 1):

Clinician educational meetings/training and educational materials: training sessions (45-minute face-to-face training sessions covering six topics); and four educational resource booklets for clinicians (one for each of the four behaviours).

Enabling resources and prompts for clinicians (staff activation): paper-based assessment tool (for clinicians to assess client risk); referral forms (paper-based referral forms for NSW Get Healthy Service and Quitline); client handout resources (paper-based resources that could be given to clients by clinicians); and a clinician poster (poster for the staff meeting room).

Client activation materials: conversation cards (coloured conversation starter cards); a client poster (poster placed in the client waiting room); and a client self-assessment form (paper-based form for clients to self-assess risk).

Audit and feedback: Recording preventive care discussion and a preventive care assessment tool.

### Control condition

The control site continued to provide their usual preventive care practices and could receive the usual support provided by the health district to do so.

### Data collection and measures

**Fig. 1:**
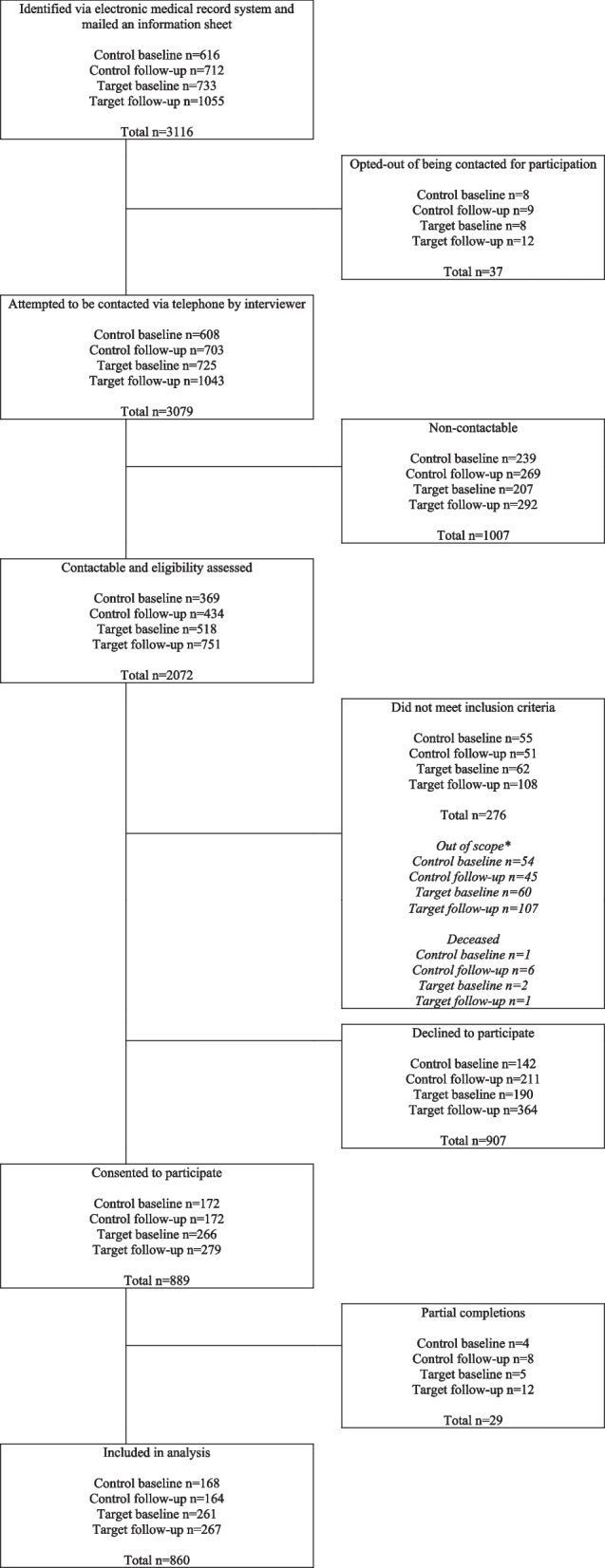
Flow diagram of client recruitment *Clients were coded out of scope if they could not recall an appointment with the community mental health service, were non-English speaking, were too sick, or were in prison, aged care, or hospital

Outcome data relating to preventive care delivery to clients was obtained via client computer-assisted telephone interviews (CATIs) administered by a trained and experienced team of telephone interviewers employed by the health district and utilised an established CATI facility and calling protocols (see Fig. 1 for participant recruitment flow). The interviewers undertaking the client surveys were not informed of which site the client had attended, although for the follow-up interviews, this would be known by the end of the survey due to the inclusion of items only asked of participants who had attended the target service. The statistician undertaking the analysis was not blinded to site allocation.

#### Client-reported data

##### Demographic and clinical characteristics

At baseline and follow-up, participants reported their gender, age, highest education level attained, employment status, marital status, and the mental health condition/s for which they had received care from the service for within the previous four months. Client postcode and the number and mode (face to face, or telephone) of service appointments within the previous four months were obtained from the electronic medical record system.

##### Health risk behaviours

At baseline and follow-up, participants reported their levels of tobacco smoking, alcohol consumption (AUDIT-C) [54], physical activity (IPAQ) [55], and fruit and vegetable intake. Survey items were based on recommended assessment tools [56]; and previous surveys undertaken with community health and mental health clients [8, 34, 43]. In line with Australian health behaviour guidelines, clients were defined as being at-risk if they reported smoking any tobacco products in the previous four months [57], consuming on average less than five serves of vegetables and/or less than two serves of fruit per day [47], consuming more than ten standard drinks on average per week or more than four standard drinks on any one occasion in the previous four months [46] or engaging in less than 150 minutes of moderate activity, or less than 75 minutes of vigorous activity, or an equivalent combination of both, per week, OR, less than two days of strength/resistance physical activity per week [44].

##### Receipt of preventive care

*Assess:* Clients were asked to report whether a clinician had assessed each of their risk behaviours, at any of their appointments during the last four months (yes/no/don’t know).

*Advice:* Clients who were classified as at risk and had reported receiving assessment were then asked for each relevant behaviour, whether their clinician advised them (at any of their appointments during the last 4 months) about the national behaviour recommendations and to modify that risk behaviour(s) (yes/no/don’t know).

*Refer:* For each behaviour where they were at-risk and had reported receiving assessment, clients were asked whether their community mental health clinician had (at any of their appointments during the last 4 months) offered them a referral to a relevant specialist or service (for example NSW Get Healthy Service, Quitline, GP, dietician or exercise physiologist, dependent upon behaviour).

#### Delivery of the implementation support strategies

Project personnel including a post-doctoral researcher and PhD student were responsible for recording the delivery of support strategies listed in Table 1. Project records consisted of a standardised spreadsheet that was updated on a consistent ‘as it occurred’ basis to log strategy delivery, which was stored on a secure drive accessible to the researchers.

### Statistical analysis

Descriptive statistics were used to summarise delivery of the implementation support strategies. Analyses were undertaken using SAS 9.4. Descriptive statistics were used to describe client demographic and clinical characteristics, health risk behaviour prevalence, and receipt of preventive care elements. Demographic variable categories were condensed as per Table 2. Dichotomous variables were calculated to define health risk behaviours (see Supplementary Table 2).

**Table 2:** Sample characteristics by group and time

**Variable**		**Control**				**Target**			
		**Baseline** **(*N* = 168)**		**Follow-up** **(*N* = 164)**		**Baseline** **(*N* = 261)**		**Follow-up (*N* = 267)**	
		***N***	**%**	***N***	**%**	***N***	**%**	***N***	**%**
Gender	Female	97	57.74	101	61.59	164	62.84	149	55.81
	Male	71	42.26	63	38.41	95	36.40	117	43.82
	Transgender or gender non-conforming	0	0.00	0	0.00	2	0.77	1	0.37
Age	18–24	22	13.10	17	10.37	43	16.48	42	15.73
	25–34	36	21.43	33	20.12	57	21.84	67	25.09
	35–44	34	20.24	39	23.78	57	21.84	51	19.10
	45–54	46	27.38	44	26.83	56	21.46	53	19.85
	55 +	30	17.86	31	18.90	48	18.39	54	20.22
Aboriginal and/or Torres Strait Islander		35	20.83	22	13.41	24^a^	8.99	24	8.99
Employment status	Employed full time	11	6.55	16	9.76	23	8.81	22	8.24
	Employed part time or casual	27	16.07	28	17.07	36	13.79	49	18.35
	Unemployed	39	23.21	36	21.95	48	18.39	56	20.97
	Can’t work — health reasons	71	42.26	57	34.76	118	45.21	93	34.83
	Other	20	11.90	27	16.46	36	13.79	46	17.23
Marital status	Never married	97	57.74	70	42.68	132	50.57	131	49.06
	Married or living together in a relationship	29	17.26	43	26.22	69	26.44	79	29.59
	Other (separated, divorced, widowed)	42	25.00	51	31.10	60	22.99	56	20.97
Education level	Some high school or less	22	13.10	22	13.41	27	10.34	28	10.49
	School certificate, Intermediate, Year 10, 4th Form	47	27.98	31	18.90	67	25.67	67	25.09
	Completed HSC, Leaving, Year 12 or 6th Form	26	15.48	27	16.46	43	16.48	38	14.23
	TAFE certificate or diploma	57	33.93	64	39.02	104	39.85	96	35.96
	University, CAE, Degree or higher	16	9.52	20	12.20	20	7.66	34	12.73
Mental health condition^c^	Depression	108	64.29	88	53.66	159	60.92	107	40.07
	Anxiety	92	54.76	82	50.00	139	53.26	95	35.58
	Schizophrenia or other psychotic disorder	41	24.40	39	23.78	82	31.42	69	25.84
	Bipolar disorder	33	19.64	36	21.95	57	21.84	51	19.10
	Personality disorder	22	13.10	17	10.37	46	17.62	33	12.36
	Post-traumatic stress disorder	23	13.69	30	18.29	39	14.94	43	16.10
	Substance use disorder	14	8.33	5	3.05	16	6.13	9	3.37
	Eating disorder	6	3.57	2	1.22	21	8.05	18	6.74
Risk status^e^	Smoking	100/168	59.52	85/164	51.83	129/261	49.43	109/267	40.82
	Alcohol overconsumption^d^	80/168	47.62	77/164	46.95	114/261	43.68	89/267	33.33
	Physical inactivity	150/168	89.29	149/164	90.85	244/261	93.49	235/267	88.01
	Inadequate fruit and vegetable intake^b^	157/162	96.91	159/162	98.15	234/240	97.50	240/249	96.39
Number of risks^f^	1	6	3.70	5	3.09	10	4.17	17	6.83
	2	43	26.54	50	30.86	76	31.67	101	40.56
	3	69	42.59	62	38.27	89	37.08	102	40.96
	4	44	27.16	44	27.16	65	27.08	29	11.65
		***M***	***SD***	***M***	***SD***	***M***	***SD***	***M***	***SD***
Appointments^a^	In person	3.5	4.3	1.5	1.8	4.4	5.9	1.5	2.9
	Telehealth	1.3	2.9	2.6	2.4	2.2	3.8	4	3.9
	Total	4.8	6.6	4	3.5	6.5	8.5	5.5	5.5

^a^Mean number of appointments during the previous 4 months to interview

^b^Participants who reported an eating disorder did not have fruit and vegetable intake measured

^c^Participants could elect multiple responses for mental health condition

^d^Participants were considered at risk for alcohol overconsumption if they consumed more than 10 standard drinks per week (chronic risk) or more than 4 standard drinks per day (acute risk)

^e^Participants who were coded at-risk when they responded ‘don’t know’ ranged from* n* = 0 (physical activity at baseline and smoking at baseline and follow-up) to *n* = 8 (inadequate fruit and vegetable intake at follow-up)

^f^*N* = 1 participant had zero risk behaviours (control follow-up)

Four dichotomous (yes/no) primary outcome variables were created: (1) client receipt of assessment for all four behaviours (regardless of risk status); (2) of those who had received assessment and were classified as at-risk for a behaviour, receipt of advice for relevant risk behaviours; (3) of those who had received assessment and were classified as at-risk for a behaviour, receipt of at least one referral offer for a relevant risk behaviour; and (4) a composite variable, ‘complete care’, reflecting receipt of all three preceding primary outcome variables. Twelve secondary outcomes for preventive care receipt were: assessed for each behaviour (4 outcome variables); advice for each at-risk behaviour they were assessed for (4 outcome variables); and received a referral offer for each at-risk behaviour they were assessed for (4 outcome variables) (Supplementary Table 1).

Logistic regression models utilising a group-by-time interaction term were developed for each of the outcome variables (16 models: 4 primary outcomes, 12 secondary outcomes; Table 2) to examine change in care delivery from baseline to follow-up in the target compared to the control group. The interaction term assessed whether the change over time is different between the groups. Change in care delivery was determined to be significantly different between groups if the adjusted *p*-value for the group by-time interaction term was < 0.05 in the regression model, and ORs were also calculated to compare the odds of care delivery between target and control groups at follow-up. Logistic regression models were adjusted for three continuous variables: age, telehealth appointment counts, and in-person appointment count during the previous four months to participation. These variables were selected as they could act as confounders to the receipt of preventive care, as identified in the existing literature [10, 32, 34, 43]. Appointment counts were particularly relevant due to changes in service care delivery from baseline to follow-up as a result of COVID-19 restrictions. Sensitivity analyses were conducted using logistic regression models (16 outcomes) that excluded repeat participants to account for not having independent observations in the sample. Unadjusted logistic regression models were run within groups between time points (baseline and follow-up) for each outcome to assess within-group changes in care delivery and were determined to be significantly different within groups if the *p*-value was < 0.05.

## Results

### Sample characteristics

Of the 3116 clients selected to participate, 2072 were contactable and hence able to be assessed for eligibility (66.5%). Of the 1796 eligible potential participants (contactable and eligibility assessed minus those who did not meet the inclusion criteria), 889 (49.5%) consented to participate, with 860 included in the analysis (baseline *n* = 429, follow-up *n* = 431) (Fig. 1). Sample characteristics are presented in Table 2. The most prevalent mental health condition was depression at baseline (control 64.3%; target 60.9%) and at follow-up (control 53.7%; target 40.1%). The most prevalent risk behaviour was inadequate fruit and vegetable intake at baseline (control 96.9%; target 97.5.1%) and at follow-up (control 98.2%; target 96.4.1%), and the least prevalent risk behaviour was alcohol overconsumption at baseline (control 47.6%; target 43.7%) and at follow-up (control 47.0%; target 33.3%).

### Effectiveness on preventive care levels

#### Between-group effects

There were no significant differential changes over time between groups for any of the primary or secondary outcomes (Table 3). The sensitivity analyses excluded n = 80 participants (control *n* = 25; target *n* = 55) who completed both surveys and demonstrated similar results to the models using the entire sample, with no new significant differential changes.

**Table 3:** Adjusted logistic regression models for primary and secondary outcomes

	Control				Intervention				Differential **adjusted**^a^ effect Odds ratio (95% CI)	*p*-value
	Baseline		Follow-up		Baseline		Follow-up			
	*N*	%	*N*	%	*N*	%	*N*	%		
**Primary outcomes**										
Assessed for all behaviours	*28/168*	16.67	*19/164*	11.59	*65/261*	24.90	*53/267*	19.85	OR = 1.19 [0.55–2.57]	0.65
Advised for all relevant risk behaviours	*11/168*	6.55	*8/163*	4.91	*28/261*	10.73	*23/266*	8.65	OR = 1.18 [0.39–3.61]	0.77
Referred for any relevant risk behaviour	*33/168*	19.64	*33/163*	20.25	*52/261*	19.92	*47/266*	17.67	OR = 0.80 [0.40–1.63]	0.55
Complete care	*7/168*	4.17	*3/163*	1.84	*10/261*	3.83	*14/266*	5.26	OR = 3.11 [0.62–15.63]	0.17
**Secondary outcomes**										
Assessed										
Smoking	*120/168*	71.43	*106/164*	64.63	*184/261*	70.50	*170/267*	63.67	OR = 1.06 [0.58–1.95]	0.85
Nutrition	*37/162*	22.84	*33/162*	20.37	*87/240*	36.25	*72/249*	28.92	OR = 0.83 [0.43–1.63]	0.59
Alcohol	*118/168*	70.24	*107/164*	65.24	*187/261*	71.65	*178/267*	66.67	OR = 0.91 [0.50–1.67]	0.77
Physical activity	*78/168*	46.43	*71/164*	43.29	*149/261*	57.09	*142/267*	53.18	OR = 1.13 [0.64–2.00]	0.66
Advised (and assessed)										
Smoking	*52/100*	52.00	*43/85*	50.59	*61/129*	47.29	*48/109*	44.04	OR = 0.87 [0.39–1.93]	0.73
Nutrition	*32/157*	20.38	*18/159*	11.32	*62/234*	26.50	*49/240*	20.42	OR = 1.47 [0.67–3.20]	0.33
Alcohol	*26/80*	32.50	*31/77*	40.26	*36/114*	31.58	*30/89*	33.71	OR = 0.76 [0.31–1.87]	0.55
Physical activity	*34/150*	22.67	*36/149*	24.16	*81/244*	33.20	*71/235*	30.21	OR = 0.87 [0.44–1.71]	0.68
Referred (and assessed)										
Smoking	*12/100*	12.00	*15/85*	17.65	*20/129*	15.50	*15/109*	13.76	OR = 0.44 [0.14–1.38]	0.16
Nutrition	*11/157*	7.01	*10/159*	6.29	*18/234*	7.69	*27/240*	11.25	OR = 1.65 [0.54–5.00]	0.38
Alcohol	*8/080*	10.00	*16/77*	20.78	*14/114*	12.28	*8/089*	8.99	OR = 0.31 [0.08–1.13]	0.07
Physical activity	*12/150*	8.00	*8/149*	5.37	*19/244*	7.79	*14/235*	5.96	OR = 1.07 [0.32–3.55]	0.91

^a^Adjusted for three continuous variables: age, telehealth appointment count and in-person appointment count

#### Within-group effects

At each site, there was one significant within-group effect from baseline to follow-up. Participants were less likely to receive nutrition assessment at follow-up compared to baseline in the target site (OR = 0.61; [0.42–0.87]; *P* = 0.01) and less likely to receive nutrition advice in the control site (OR = 0.54; [0.29–1.00]; *P* = 0.05) (Supplementary Table 3).

### Implementation support package delivery and fidelity

The delivery of the implementation support strategies is summarised in Table 1. Of the four strategies planned for delivery, one was delivered as intended (clinician training and educational resources), two were delivered but not as originally intended (enabling resources and prompts for clinicians, and client activation materials), and one was not delivered (audit and feedback). Regarding the strategy delivered as intended (clinician training and educational resources), nineteen to thirty staff attended each training session which occurred in the first six weeks of the implementation period, and delivery of the educational resources took a staged approach and occurred in the following weeks (7, 8 and 10). The two strategies delivered to some extent (clinician enabling resources and client activation materials), although provided to the service, had poor uptake due to COVID-19 protocols and late-stage implementation (see footnotes Table 1). For example, the client activation conversation cards were removed from the waiting room and staff were advised to not provide shared hardcopy materials to clients (including the clipboard and pen for the client self-assessment form). Furthermore, the enabling resources were delivered at the end of the implementation period, which limited their opportunity for use. For example, the poster in the staff meeting room to prompt preventive care would have had limited visibility as staff review meetings were reduced when COVID-19 restrictions took effect. The audit and feedback strategy, although identified as appropriate by staff, was unable to be implemented due to service changes and COVID-19 limitations. Firstly, service changes included the health district-wide reformatting of clinical case review forms so that individual services were no longer able to amend the forms for their own purposes, including the changes that were to be made as part of the support package to enable the collection of preventive care provision data during review meetings. Secondly, there was a significant reduction of review meetings to avoid face-to-face contact and align with COVID-19 protocols.

## Discussion

To the author’s knowledge, this is the first study to examine the effectiveness of an implementation support package designed using a participatory approach that aimed to increase preventive care delivery for multiple health risk behaviours by community mental health services. The four month implementation support package was ineffective in increasing the four primary outcome measures of (1) assess for all behaviours, (2) advice for all relevant risk behaviours, (3) refer for any relevant risk behaviour, and (4) complete care, nor the 12 secondary outcome measures. The implementation support package was not delivered as planned; with three of four final strategies delivered, of which two were not implemented as intended due to contextual variables including the impact of the COVID-19 pandemic on service delivery. Hence, findings should be interpreted within this context.

It is likely that the limited fidelity in delivery of support strategies impacted overall effectiveness. The two components within the clinician training and education strategy were delivered as intended. During the implementation period, the training sessions occurred within the first six weeks and the educational resources were delivered consistently. However, other strategies were partially delivered, delivered late, or not delivered at all due to a number of issues, including those associated with the emerging COVID-19 pandemic such as state-wide health service changes and difficulty engaging with services. Existing research literature would suggest it is likely that the limited success of the implementation support package in the current study is at least partly due to these strategies not being delivered as intended [58–60].

Although it is not possible to determine the impact of the emerging COVID-19 pandemic on fidelity of support strategy delivery, or on staff capacity to provide preventive care, it is likely to have had an impact. The first COVID-19 pandemic wave triggered federal and state governments to implement guidelines limiting face-to-face contact between staff and specifically requesting that health facilities limit in-person contact with clients. These changes came into effect during the end of the implementation period and remained during follow-up data collection. The changes to clinical service provision leading up to and during the COVID-19 lockdown placed unprecedented strain on the health system due to factors such as wearing masks, general concerns about occupational health risks and the widespread implementation of new telehealth systems and protocols. Staff absenteeism increased due to COVID-19 protocols including non-attendance at work with respiratory symptoms, abiding by stay-at-home orders due to close contact (usually children or other family members), or testing positive to COVID-19, and a small number of clinicians were redeployed to man COVID-19 call centres which placed a greater burden on remaining clinicians. For example, service data collected in 2020 between the end of March and the start of July showed 26 staff took sick leave related to COVID-19 testing and isolation protocols resulting in a total of 136 days ineligible to work (including admin and clinical staff). Each staff member was additionally absent for four hours for vaccinations.

Furthermore, services also moved to telehealth methods of service delivery wherever possible, a significant change to practice. Clinical records indicate a substantial reduction in in-person appointments and appointments overall was experienced by clients in aim of limiting cross-infection (Table 2), a finding reflective of the wider NSW healthcare system [61]. Appointments needed to include COVID-19 screening questions and advice regarding vaccines and testing thus reducing the time available for other care provision. This reduction may have meant there were less opportunities for care which may have affected preventive care delivery. COVID-19 increased the side effects of many common psychiatric medications and as a result, clinicians needed to focus on modifying dosage particularly for antipsychotics due to the risk of worsening mental illness. Clinicians may have been more likely to prioritise the provision of acute care due to the deteriorating mental health of clients [62, 63]. The observed reductions in some preventive care elements in both the target (nutrition assessment) and control sites (nutrition advice) may have been contributed to by such factors (Supplementary Table 3). Studies indicate mental health staff face competing clinical priorities as a barrier to providing preventive care generally [64, 65] and such a barrier may have been heightened during COVID-19.

There have been calls to publish more explicit accounts regarding the involvement of end-users in health service research [66–68]. This study describes the process and outcomes of a two-phase participatory approach to design a support package consisting of multiple implementation strategies for community mental health service clinicians, which may aid knowledge translation and evaluation of participatory methods [69, 70]. A participatory approach involving mental health clinicians and managers was utilised to design implementation strategies to address clinical, professional, cultural, and organisational factors that distinguish community mental health service delivery from other community health services [65, 71, 72].

The current study engaged in a two-phase participatory design process which involved a smaller and more senior working group to identify challenges and potential solutions (e.g. clinician training and educational resources), followed by involvement with a larger group of all clinicians to provide feedback on outcomes from the working group (e.g. details and content of training and educational resources). It is possible this process did not adequately capture the needs and wants of clinicians ‘on the ground’ and tailor strategies accordingly. The participatory design process occurred over a total of seven months, including six meetings over five months with the implementation working group of senior staff (phase one), and three meetings over two months with on-the-ground clinicians (phase two), followed immediately by the delivery of the four month implementation support package. Such timing may not be sufficient, particularly with limited time and opportunity to develop the implementation strategies between the design phases. This meant several strategies were delivered later than intended during the implementation period and ultimately were more severely impacted by COVID-19. Furthermore, the barriers to preventive care provision were likely to have changed between the participatory design process which was undertaken prior to the pandemic emergence and full implementation due to the rapidly changing context of COVID-19. An understanding of the context of preventive care delivery in community mental health services gained from the participatory process used may not have been sufficient to select effective implementation strategies, and a more systematic and rigorous method of participatory research design, such as co-design workshops, may be beneficial [68].

In phase two, clinicians requested more focus of the implementation strategies on how to provide support to clients in line with the ‘assist’ component of the 5As (discussion of the benefits and barriers to change, providing counselling to change behaviours (such as motivational interviewing), and/or providing additional supports including pharmacotherapy, educational materials or self-help materials) [27, 73], which could suggest that clinicians would like to improve their skills in preventive care delivery to provide greater behaviour change support to their clients, instead of, or in addition to, referring on to other services. As such, the education and training package included a large amount of content on motivational interviewing, conversation starters, hints and tips for behaviour change and practical resources to offer clients. Other literature supports this, with studies demonstrating that staff working in community-based mental health settings want to assist mental health clients in making behaviour changes [74].

As previously discussed, not all implementation strategies were delivered as intended, a finding that may have contributed to the absence of measured impact. For example, there is evidence for the potential of client activation materials [75] however this strategy had limited fidelity in the current study largely due to changes in service protocols and restrictions resulting from COVID-19. Future research should implement and measure the suitability and effectiveness of client activation materials in community mental health settings. Fidelity could potentially be improved in future research with implementation support strategies that are robust and are designed within the context on virtual staff training and care delivery. For example, by employing an implementation support officer or clinical champions in a dedicated role to reduce the burden on mental health staff to deliver certain strategies, and to reinforce the importance of preventive care delivery and provide assistance and support to clinicians. Future research should consider involving clients in content development [76]. Overall, achieving improved fidelity requires sufficient resourcing for delivery of implementation strategies, as well as a high level of planning in which commitment from all stakeholders in the clinical setting should be negotiated.

## Limitations

With regard to limitations, the design of the current study included two sites (one target and one control) which meant the implementation support package was delivered in one community mental health service in a single health district, thereby potentially limiting its generalisability to other districts and jurisdictions. The lack of randomisation for the two sites may have compromised the ability to estimate probabilities of differences due to potential confounding factors. However, randomisation of sites in community trials has been suggested as unacceptable for pragmatic trials within health services [77]. The lower than anticipation completion rate led the study to be underpowered, which may have contributed to the lack of significant results. The main outcome measures were all client self-report receipt of preventive care. There is an absence of research directly comparing different methods of data collection to measure preventive care delivery in mental health services (e.g. client-report, staff report and medical record audit); however, the literature indicates client report of preventive care received has strengths relative to other approaches [20, 78].

## Conclusion

The implementation trial was not effective at increasing preventive care receipt by people accessing a community mental health service; potentially contributed to by limited fidelity and impacts of COVID-19. Findings suggest there is a need to continue to understand what is required to increase preventive care delivery in community-based mental health settings. Further investigation is required to determine optimal participatory design methods to develop effective implementation support strategies that lead to increased preventive care delivery in community mental health settings, including those that support delivery of care within the ongoing context of COVID-19.

### Supplementary Information

**Additional file 1:** **Supplementary Table 1.** Outcome variables definitions.

**Additional file 2:** **Supplementary Table 2.** Risk measures and definitions.

**Additional file 3:** **Supplementary Table 3.** Descriptive within group differences.

## Data Availability

The datasets generated and/or analysed during the current study are not publicly available due to protecting the confidentiality of study participants but are available from the corresponding author on reasonable request.

## Abbreviations

AAR: Ask-advice-refer
5As: Ask-advise-assess-assist-arrange
GP: General practitioner
NSW: New South Wales
CATI: Computer-assisted telephone interview
AUDIT-C: Alcohol Use Disorders Identification Test
NHMRC: National Health and Medical Research Council
OR: Odds ratio
RCT: Randomised controlled trial
