# Supplementary material for: Evaluation of an implementation support package to increase community mental health clinicians’ routine delivery of preventive care for multiple health behaviours: a non-randomised controlled trial
Source: Implement Sci Commun. 2023 Nov 13;4:137. doi: 10.1186/s43058-023-00509-0 (PMC10644601; doi:10.1186/s43058-023-00509-0)
Supplement: Supplementary file 1 — Additional file 1: Supplementary Table 1. Outcome variables definitions. [file 43058_2023_509_MOESM1_ESM.docx]

| Supplementary Table 1. Primary and secondary outcome variable definitions | |
| --- | --- |
| **Outcome variable** | **Definition** |
| **Primary outcomes** |  |
| Assessed for all behaviours | client was assessed for all four health behaviours (yes vs no) |
| Advised for all risk behaviours | client received advice for all at-risk behaviours they were assessed for (yes vs no) |
| Referred for any risk behaviour | client received at least one referral for an at-risk behaviour they were assessed for (yes vs no) |
| Complete care | client was assessed for all four health behaviours, received advice for all at-risk behaviours they were assessed for, and was offered at least one referral for an at-risk behaviour they were assessed for (yes vs no) |
| **Secondary outcomes** |  |
| Assessed |  |
| Smoking | client was assessed for whether they smoke (yes vs no) |
| Alcohol | client was assessed for how much alcohol they drink (yes vs no) |
| Nutrition | client was assessed for how many serves of fruit they usually eat each day, or, how many serves of vegetables they usually eat each day (yes vs no) |
| Physical Activity | client was assessed for how many days per week they do physical activity, or, what type of physical activity they do (yes vs no) |
| Advised (and assessed) |  |
| Smoking | client received advice to reduce or quit their smoking (yes vs no) |
| Alcohol | client received advice about the levels of alcohol consumption which reduce the risk of alcohol-related harm, or advised to make any changes to the amount of alcohol they drink (yes vs no) |
| Nutrition | client received advice about the recommended amount of fruit and/or vegetable to eat, or advised to make any changes to their fruit and/or vegetable intake (yes vs no) |
| Physical Activity | client received advice about the recommended amount or types of physical activity or advised to make any changes to their physical activity levels (yes vs no) |
| Referred (and assessed) |  |
| Smoking | client reported being offered a referral to a specialist or service that might help reduce the amount they smoke, or quit (yes vs no) |
| Alcohol | client reported being offered a referral to a specialist or service that might help modify their alcohol consumption (yes vs no) |
| Nutrition | client reported being offered a referral to another clinician or service to help improve their fruit and vegetable intake, or diet more broadly (yes vs no) |
| Physical Activity | client reported being offered a referral to a specialist or service that might help improve their physical activity levels (yes vs no) |

*Participants who responded ‘don’t know’ were classified as not having received care
