# Supplementary material for: Evaluation of an implementation support package to increase community mental health clinicians’ routine delivery of preventive care for multiple health behaviours: a non-randomised controlled trial
Source: Implement Sci Commun. 2023 Nov 13;4:137. doi: 10.1186/s43058-023-00509-0 (PMC10644601; doi:10.1186/s43058-023-00509-0)
Supplement: Supplementary file 2 — Additional file 2: Supplementary Table 2. Risk measures and definitions. [file 43058_2023_509_MOESM2_ESM.docx]

| Supplementary Table 2. Risk measures and definition of ‘at risk’ variables included in outcomes | | |
| --- | --- | --- |
| **Risk Variable** | **Items [*response options*]^a^** | **Definition of ‘at risk’^b^** |
| Tobacco smoking | How often they currently smoke cigarettes or any other type of tobacco product  [*daily; at least once a week; less than once a week; not at all (quit less than 4   months ago); not at all (quit 4 months or more ago); never smoked*] | Smoked in the last four months,   or quit within the last four months |
| Harmful alcohol consumption | How often do you have a drink containing alcohol?  [*never, monthly, or less, 2-4 times a month, 2-3 times a week, 4+ times a week*]    How many days a week do you have a drink containing alcohol?  [*0-7 days*]    How many standard drinks would you have on a typical drinking day? A standard drink is 1 schooner of light beer, a middy of full strength beer, a 100ml glass of wine or a 30ml nip of spirits.  [*1 or 2; 3 or 4; 5 or 6; 7 to 9; 10+*]  How often would you have five or more standard drinks on one occasion?  [*never; less than monthly; monthly; weekly; daily or almost daily*] | >10 standard drinks/week or >4 standard drinks/day on any day |
| Inadequate fruit & vegetable intake | How many serves of fruit do you usually eat each day at the moment? A serve is 1 medium piece, 2 small pieces of fruit or 1 cup of diced pieces.  [*0; 1; 2; 3; 4; 5; 6 or more*]  How many serves of vegetables do you usually eat each day at the moment? A serve is half a cup of cooked vegetables or 1 cup of salad vegetables  [*0; 1; 2; 3; 4; 5; 6 or more*] | <2 serves/day of fruit or <5 serves/day of veg |
| Inadequate physical activity | During the LAST 7 DAYS, on HOW MANY DAYS did you do VIGOROUS physical activities, for at least 10 minutes at a time? (e.g. running, jogging, gym classes, boxing, soccer or squash)  [*0 –7 days*]  On average, how many MINUTES PER SESSION did you spend on those VIGOROUS physical activities?    During the LAST 7 DAYS, on HOW MANY DAYS did you do MODERATE physical activities, for at least 10 minutes at a time? (e.g. fast walking, baseball, tennis, easy bicycling, volleyball, easy swimming)  [*0 –7 days*]  On average, how many MINUTES PER SESSION did you spend on those MODERATE physical activities?    During the LAST 7 DAYS, on HOW MANY DAYS did you do any type of muscle  strengthening activities such as exercises using free weights, body weight exercises or gym-based strength exercises? (e.g. exercises using free weights, body weight exercises or gym-based strength exercises).  [*0 –7 days*] | <150 min moderate activity or,   <75 min vigorous activity or,   less than an equivalent combination of both^c^ or,  <2 days/week of including strength/resistance in physical activity |

^a^All items included a ‘don’t know’ option.

^b^Participants who responded ‘don’t know’ to corresponding survey measures defining each dichotomised health behaviour were considered at risk.

^c^An equivalent combination of moderate and vigorous activity was calculated by dividing the number of moderate activity minutes per week by two, then adding the number of vigorous activity minutes per week. If this total was less than 75, then considered inadequate (i.e. risk).
