# Supplementary material for: Evaluation of an implementation support package to increase community mental health clinicians’ routine delivery of preventive care for multiple health behaviours: a non-randomised controlled trial
Source: Implement Sci Commun. 2023 Nov 13;4:137. doi: 10.1186/s43058-023-00509-0 (PMC10644601; doi:10.1186/s43058-023-00509-0)
Supplement: Supplementary file 3 — Additional file 3: Supplementary Table 3. Descriptive within group differences. [file 43058_2023_509_MOESM3_ESM.docx]

| Supplementary Table 3. Descriptive within group differences | | |
| --- | --- | --- |
|  | Difference (%) within groups from baseline to follow-up | |
|  | Control | Target |
| **Primary outcomes** |  |  |
| Assessed for all behaviours | ↓5.08% | ↓5.05% |
| Advised for all relevant risk behaviours | ↓1.64% | ↓2.08% |
| Referred for any relevant risk behaviour | ↑0.61% | ↓2.25% |
| Complete care | ↓2.33% | ↑1.43% |
| **Secondary outcomes** |  |  |
| Assessed |  |  |
| Smoking | ↓6.80% | ↓6.83% |
| Nutrition | ↓2.47% | ↓**7.33%** |
| Alcohol | ↓5.00% | ↓4.98% |
| Physical Activity | ↓3.14% | ↓3.91% |
| Advised (and assessed) |  |  |
| Smoking | ↓1.41% | ↓3.25% |
| Nutrition | ↓**9.06%** | ↓6.08% |
| Alcohol | ↑7.76% | ↑2.13% |
| Physical Activity | ↑1.49% | ↓2.99% |
| Referred (and assessed) |  |  |
| Smoking | ↑5.65% | ↓1.74% |
| Nutrition | ↓0.72% | ↑3.56% |
| Alcohol | ↑10.78% | ↓3.29% |
| Physical Activity | ↓2.63% | ↓1.83% |

Bolded percentages indicate statistically significant p-value (<0.05)

↓decreased at follow-up from baseline

↑increased at follow-up from baseline
